# Midlife Work and Women’s Long-Term Health and Mortality

**DOI:** 10.1007/s13524-019-00839-6

**Published:** 2019-12-11

**Authors:** Jennifer Caputo, Eliza K. Pavalko, Melissa A. Hardy

**Affiliations:** 1 Max Planck Institute for Demographic Research, Konrad-Zuse-Straße 1, 18057 Rostock, Germany; 2 Department of Sociology, Indiana University, Bloomington, IN USA; 3 Department of Sociology and Criminology, Pennsylvania State University, University Park, PA USA

**Keywords:** Work, Work experiences, Health, Mortality, Women

## Abstract

Although paid work is a well-established predictor of health, several gaps in our knowledge about the relationship between adult work patterns and later health and mortality remain, including whether these benefits persist over long periods and whether they are dependent on subjective experiences with work. We draw on more than three decades of data from the National Longitudinal Survey of Mature Women to assess how labor force participation over a period of 20 years during midlife is related to mental and physical health and mortality over the following 16–25 years. We find that consistent work earlier in life continues to predict improved health and longevity over many years as women enter late life, and this relationship does not differ between women with positive and those with negative subjective work experiences. These findings add to knowledge about how key adult social experiences are related to health as individuals enter later life.

**Electronic supplementary material:**

The online version of this article (10.1007/s13524-019-00839-6) contains supplementary material, which is available to authorized users.

## Introduction

Paid work is one of the most important social determinants of health for both women and men, providing material and social resources that protect and enhance well-being throughout life. Research has shown that the apparent health benefits of work extend across social groups and persist at different life stages (Frech and Damaske 2012; Moore and Hayward 1990; Pavalko and Smith 1999; Raymo et al. 2013). However, three questions about the long-term relationship between adult work and health remain. First, we know relatively little about how the relationship evolves over time and whether health differences between workers and nonworkers diminish, grow, or remain stable as adults pass retirement and enter late life. This is because health is typically measured at one point in time or over a short follow-up period, even among studies using longitudinal data on work (Frech and Damaske 2012; Luoh and Herzog, 2002; Pavalko and Smith 1999). Second, knowledge about how subjective experiences with work shape the relationship between work and later health is limited despite studies showing that these experiences moderate work’s health benefits in the short term (Kivimäki and Kawachi 2015; Lennon 1994; Pavalko et al. 2003; Pugliesi 1995). It is thus unclear whether work predicts improved health even for those with consistently negative work experiences or whether the association is stronger for those with positive experiences. Third, questions about whether the relationships between work and long-term health vary by the indicator examined have been overlooked because prior research has tended to focus on physical or mental health or mortality rather than considering these patterns simultaneously. Work experiences that manifest health effects via more direct and proximate pathways, such as through physical and mental health, may not affect more distant outcomes, such as longevity.

In this study, we draw on more than three decades of longitudinal data from the National Longitudinal Survey of Mature Women (NLS-MW) to assess how 20 years of women’s midlife work experiences are related to long-term health and mortality over the following 16–25 years as they pass retirement and enter late life. Recognizing that there are intertwining pathways through which this process emerges, our analysis makes use of three outcomes that may capture more proximate and immediate effects of social experiences (with depressive symptoms) as well as those that may be more secondary or emerge only over longer-term follow-up periods (with functional limitations and mortality). The NLS-MW cohort of women lived through a significant shift in the gendered nature of work in the United States, and they undoubtedly encountered multiple stressors associated with their entry into the labor force (Gershuny and Robinson 1988; Reskin and Roos 1990). Thus, our analysis contributes to knowledge about the long-term health effects of work and subjective work experiences within this context of social change.

## Background

Women’s participation in the paid workforce increased during the second half of the twentieth century. Women composed less than 30% of workers in the 1950s but nearly 47% by the end of the 1990s, with little change since (U.S. Census Bureau 2011). Almost as likely to work as their male counterparts, they are represented in increasingly diverse fields (U.S. Census Bureau 2011). A large body of sociological research has explored the various effects of this historic shift in the gendered nature of work and family life, including what it meant for the health and well-being of women in the United States. Scholars initially raised concerns that women’s movement into the labor force would result in stress by pitting family and work demands against each other (e.g., Sorensen and Verbrugge 1987; Waldron and Jacobs 1988; Weatherall et al. 1994). However, results from the majority of these and other studies showed that women who worked were better off mentally and physically than nonworkers, including those with husbands and young children—a pattern that continues to be replicated (Frech and Damaske 2012; Klumb and Lampert 2004; Leupp 2017; Pavalko et al. 2007; Schnittker 2007). Social scientists explain the consistent positive relationship between work and health by pointing to the many material, social, and psychosocial resources that employment provides, from income and health insurance to supportive relationships and feelings of self-worth (Burgard and Lin 2013; Moen 1996; Pavalko and Smith 1999; Repetti et al. 1989).

As scholars have frequently noted, the relationship between health and work is reciprocal, complicating the ability to make causal arguments. However, although longitudinal studies have shown a selection effect whereby happier and healthier women are more likely to work, the positive relationship between work and health remains even after this selection process is accounted for (for a review, see Klumb and Lampert 2004). For example, in an earlier longitudinal analysis based on the NLS-MW, Pavalko and Smith (1999) found that work measured at several points in midlife predicted better physical and mental health shortly later. More recently, Frech and Damaske (2012) showed that stably working women were healthier at age 40 even after factors that selected them into different pathways were accounted for. Analyses of register data in the European context further support causal claims about the work and health relationship (e.g., Lundin et al. 2010; Montez et al. 2014). Research suggests that workers (both male and female) may even live longer than nonworkers (Hibbard and Pope 1991; Luoh and Herzog 2002; Montez et al. 2014). These and other findings provide powerful evidence that participation in paid work has real advantages for well-being. Even so, three distinct gaps in our knowledge about the relationship between adult work and health over the life course remain.

First, it is unclear whether and how the relationship between adult work histories and later health changes as individuals retire and enter late life. Studies have shown that the stability and consistency of adult work are associated with well-being, leading scholars to hypothesize that the longer individuals were in the workforce, the greater their access to health-linked resources both during and after employment (Frech and Damaske 2012; Moore and Hayward 1990; Pavalko and Smith 1999; Raymo et al. 2013). However, even studies making use of longitudinal data on work histories tended to measure later health at only one follow-up period (e.g., Frech and Damaske 2012; Pavalko and Smith 1999). Supporting the need to look at how these patterns play out over long-term follow-up periods, Willson et al. (2007) found that socioeconomic resources predicted diverging health trajectories over a 16-year period. Because studies have yet to investigate parallel questions about the relationship between work and health at multiple follow-up points or over extended periods, it is unclear whether it follows a similar pattern of increasing disparity or, alternatively, whether the association remains stable or decreases as adults encounter later-life health challenges.

A second gap stems from relative inattention to subjective experiences with and perceptions of work as factors implicated in this relationship. As noted earlier, work is hypothesized to improve health not only because it provides structural and material resources but also because it offers supportive ties, a sense of purpose, and self-worth (Burgard and Lin 2013; Moen 1996; Repetti et al. 1989). Some prior research has explored how certain structural aspects of jobs are related to mortality, finding that “bad” jobs providing lower incomes, pensions, and insurance (Burgard and Lin 2013; Hayward and Grady 1990; Johnson et al. 1999; Raymo et al. 2013) as well as more hazardous and less complex jobs (Moore and Hayward 1990) decrease and may even reverse the longevity advantages associated with stable work. Additionally, cross-sectional and short-term studies have reliably found that work appraisals and experiences moderate the relationship between work and health (Kivimäki and Kawachi 2015; Lennon 1994; Pavalko et al. 2003, 2007; Pugliesi 1995). For example, Kivimäki and Kawachi’s (2015) review showed that individuals experiencing work stress had up to 40% greater risk of a heart incident or stroke. Pavalko et al. (2007) found that women with high work commitment had fewer functional limitations. Despite these findings, it is unclear whether these subjective experiences and appraisals also impact the health–work relationship in the long term. Health-linked rewards of paid work may emerge for those with positive work experiences but not for those with negative work appraisals. These questions may be especially important to explore among cohorts of female workers who entered work during the 1960s–1980s and undoubtedly encountered some resistance and discriminatory behavior (e.g., Jacobs 1989; Reskin and Roos 1990). Additionally, although women are increasingly represented in traditionally male-dominated fields, the types of work available to women during the mid-century were limited and often lower in status, possibly increasing the likelihood of negative experiences (Reskin and Roos 1990; U.S. Census Bureau 2011).

A third question is whether the relationship between work and health depends on the health dimension examined. Aging and life course scholarship stresses that the long-term health effects of earlier experiences emerge from intertwined and interactive social, psychosocial, and biological processes (Kuh et al. 2003; Lynch and Smith 2005; Springer 2009). These insights highlight the strengths of research designs that rely on a range of health outcomes. For example, Springer (2009) found that abusive experiences in childhood predicted worse adult health via several linked mental and physical health outcomes. As health psychologists have noted, negative social experiences may evoke stress hormones that impact physical health directly as well as via feelings of depression and anxiety, creating increased risk for physical health problems, such as cardiovascular or respiratory disease (e.g., Cohen and Rodriguez 1995; Taylor et al. 1997). Distressing experiences may also be related to health through their impact on coping mechanisms or health behaviors. Hence, although social experiences may have the most robust effects on mental health, they also clearly have the potential to “get under the skin” and are related to physical health outcomes and even mortality (Aneshensel et al. 1984; Hayward and Gorman 2004; Katon and Ciechanowski 2002; Taylor et al. 1997). Medical sociologists have underscored the need to consider diverse outcomes for decades, stressing that social groups may express feelings of distress in different ways (e.g., Aneshensel 1992; Williams 1997). Including multiple outcomes in a longitudinal analysis of health trajectories can not only capture diverse reactions to stress but also detect proximate as well as more delayed outcomes that may result from cumulative or iterative processes, emerging only over longer periods. To date, research on work and health has tended to focus on a single health dimension rather than consider several different health outcomes.

## Research Questions

Our analyses are guided by the following questions:

1. How is midlife work related to mental and physical health and mortality in later life, and are these relationships stable over time?
2. Do subjective work experiences and appraisals of work—including job commitment, attitude, and perceptions of discrimination—moderate the relationship between midlife work and later health and mortality?
3. Do these patterns vary by the type of indicator examined (including depressive symptoms, functional limitations, and mortality)?

In response to the first research question, we expect that women who work consistently will have better health and live longer than their counterparts who did not work during this period, consistent with the majority of previous research (e.g., Frech and Damaske 2012; Pavalko and Smith 1999; Ross and Mirowsky 1995; Waldron and Jacobs 1988). However, the literature provides conflicting expectations for how these patterns will play out over time. On the one hand, the advantages of work may diminish as time wears on and as women enter later life and encounter many other situations and experiences that influence health. On the other hand, health differences between women who worked and those who did not may grow as they age. Of course, it is also possible that the negative effects of not working will remain stable and persistent no matter how long the observation window.

Addressing the second research question, we predict that women with predominantly positive work experiences (including never experiencing discrimination, and liking and being committed to their jobs throughout midlife) will have the best health and live the longest relative to women who do not work, whereas women with negative midlife work experiences may not have better long-term health than nonworking women. These expectations are guided by studies showing that stressful work experiences and poor evaluations of work are associated with decreased well-being (e.g., House et al. 1979; Lennon 1994; Pavalko et al. 2003; Pugliesi 1995).

Last, we expect variation in how these patterns play out across the three health outcomes. In particular, drawing on scholarship suggesting that social experiences have more direct effects on relatively proximate outcomes (e.g., Cohen and Rodriguez 1995; Springer 2009; Taylor et al. 1997), we expect that midlife work and work experiences will reliably predict mental and physical health but that the association with later-life mortality will be weaker.

## Data and Method

### Sample

**Fig. 1:**
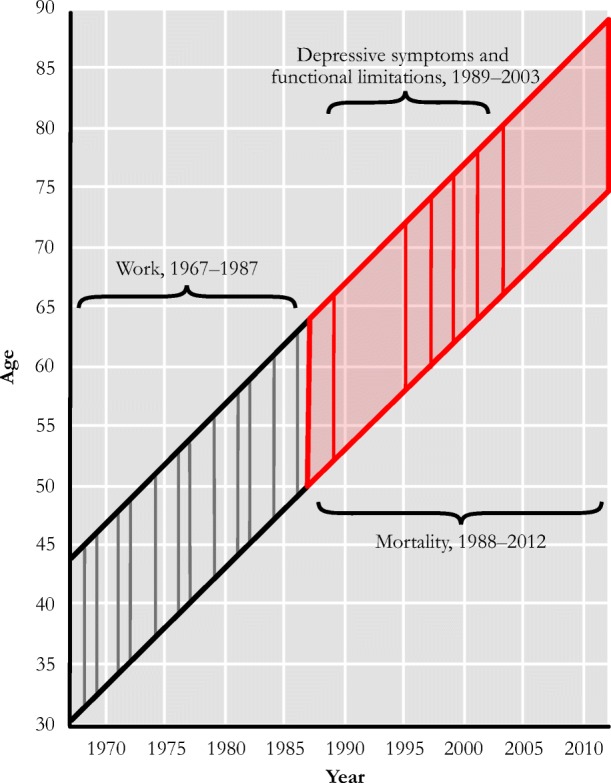
Longitudinal design

The National Longitudinal Survey of Mature Women (NLS-MW) is a nationally representative sample of 5,083 women aged 30–44 in 1967. This cohort of women was thus in their prime adult working years during the period in U.S. history when women’s participation in the labor force increased most sharply (U.S. Department of Labor 2015). The NLS-MW sample was reinterviewed regularly approximately every two years until 2003, resulting in a total of 36 years of data on work and family life. Our analysis summarizes information on women’s work over the first 20 years of the study until they were 50–64 years old in 1987 and assesses the relationship between these midlife work histories and their mental and physical health during the following 16 years (to 2003) as well as mortality, last updated in 2012. This longitudinal design is modeled in Fig. 1, which shows the age ranges of women over the study period. Labels denote the time frames during which work and health are assessed, with vertical lines indicating the specific survey waves.

Response rates were strong. For each of the 13 follow-up waves to 1989, between 95% and 98% of the living sample that had participated in the previous wave was successfully reinterviewed. Even so, by 1989 (the wave during which detailed data on health were first collected), attrition had reduced the sample to 3,353 women, 73.2% of the 4,579 women who were still living at this time. The sample included enough African American women to allow for comparisons with White women, but the very few women in the sample who belonged to any other racial or ethnic group were excluded from our analysis (*N* = 45), leaving 3,308 respondents. Because of the total of 21 separate data points, we are able to replace year-specific missing data on most independent variables with data from the most recent completed interview. With further restrictions due to missing cases on self-rated health in 1967 (*N* = 64) as well as a few cases of cross-wave missing data on education (*N* = 9) and income (*N* = 28), our analytic sample size is 3,207 for mortality, nearly 96% of the sample interviewed after 1987. To increase comparability across the two health outcomes, we limit the analyses for depressive symptoms and functional limitations to women with nonmissing information on a majority of the items during at least two of the six points of data collection for both, resulting in an analytic sample size of 2,781.

As with any panel study, attrition in NLS-MW creates selection concerns given that women who died before 1989 or dropped out of the study for other reasons are not in our sample. Table 1 compares the baseline characteristics of the analytic and attrited samples. Of the 1,652 African American and White age-eligible women in the baseline sample who were never reinterviewed after 1987, 19.79% (*N* = 327) had died. The other 1,325 women refused to be interviewed or were not located. Based on one earlier dichotomous measure of depressed affect in 1981, women excluded from the analysis were no more likely to report being depressed. However, both women who died and women who attrited for other reasons were more likely to report health limitations and lower self-rated health at baseline in 1967. The attrited sample was also slightly older, more likely to be African American, less likely to have a college degree, more likely to have lower income, and less likely to be married and more likely to be divorced at baseline. Women who left the sample were also less likely to report working for pay from 1967 to 1987. These differences mean that healthier and more advantaged women were more likely to stay in the study, which may inflate estimates of the relationship between work and health.

**Table 1:** Comparison of analytic and attrited samples

	Analytic Sample (*N* = 3,207)	Total Attrited Sample (*N* = 1,652)
Died by 1987 (%)	0.00	19.79*
Depressed Affect, 1981 (%)	32.28	34.73
Health Prevented or Limited Work, 1967 (%)	16.84	21.85*
Self-rated Health, 1967 (0–3)	2.25	2.14*
	(0.78)	(0.84)
Age, 1967 (30–44)	37.08	37.67*
	(4.36)	(4.34)
African American (%)	26.69	29.18*
Education (%)		
0–6 years of education	6.80	7.57
7–11 years of education	34.14	36.99*
12 years of education	41.97	40.86
13+ years of education	17.09	14.59*
Marital Status, 1967 (%)		
Married	87.90	83.84*
Divorced	4.33	6.42*
Widowed	2.56	3.27
Never married	5.21	6.48
Natural Log of Family Income, 1967	11.74	11.72*
	(0.14)	(0.13)
Proportion of Waves Worked, 1967–1987	.54	.48*
	(.35)	(.40)

*Note:* Standard deviations are shown in parentheses.

* Significantly different from the analytic sample, *p* < .05.

To investigate the effects of these nonrandom patterns of attrition, we conducted supplemental analyses using propensity score weights that adjusted the analytic sample relative to the attrited sample on the basis of these earlier characteristics, including health limitations, self-rated health, and depression. Analyses using the weights are shown in Table A1 in the online appendix. Because the findings are substantively the same and valid propensity weights were available only for women who had complete information on the relevant items, we present results based on the unweighted analyses.

### Measures

#### *Dependent Variables*

##### Depressive symptoms

In 1989, 1995, 1997, 1999, 2001, and 2003, respondents were asked seven questions from the Center for Epidemiologic Studies Depression scale (CES-D; Radloff 1977) to measure their mood in the past week. Specifically, respondents reported how often in the past week they (1) had been unable to shake the blues, (2) had trouble focusing on tasks, (3) felt that everything was an effort, (4) had restless sleep, (5) felt sad, (6) felt lonely, and (7) could not get going. Response categories for each item ranged from 0 (rarely) to 3 (most or all of the time), with possible CES-D scores for each year ranging from 0 to 21. The inter-item correlation of the scale in 1989 is .89.

##### Functional limitations

At the same six waves from 1989 to 2003, respondents were asked five questions assessing their functional limitations, most of which derive from the Nagi index (1976). These items ask whether a health problem caused difficulty with (1) walking; (2) using stairs; (3) sitting for long periods; (4) stooping, kneeling, or crouching; and (5) carrying heavy objects. In 1989, these questions were asked only of women who first said they had a health problem of some kind, and the response categories were yes (coded 1) or no (coded 0). After 1989, all women were asked questions about functional limitations; response categories were 0 (not at all difficult), 1 (a little difficult), 2 (somewhat difficult), and 3 (very difficult/can’t do). To make these responses consistent with the 1989 measure, we coded those reporting no or a little difficulty as 0 on each item and coded those reporting that the task was somewhat difficult or that they could not do it as 1.^1^ Relying on the assumption that respondents do not perform these activities because they cannot do them, a fifth response category in these later waves—“don’t do”—was also coded 1.^2^ Summing these items, functional limitations scores range from 0 to 5 for each year. The interitem correlation for 1989 is .75.

##### Mortality

Information about mortality was recorded by NLS-MW interviewers through the last wave of data collection in 2003. Beginning in 2009, the second and third authors worked with the Demographic Survey Division of the U.S. Census Bureau and the National Center on Health Statistics (NCHS) to match NLS-MW respondents to death certificates using Social Security numbers (SSNs), names, dates of birth, sex, and race. The NCHS’s national database of mortality data derives from state death records, and more than 94% of the sample was matched to these records based on SSNs alone. Additional mortality data for 2008–2012 come from the Social Security Death Index.

##### Work History

Our central independent variable in this analysis is women’s participation in the workforce from baseline in 1967 to 1987. The NLS-MW mainly organized women by employment status into three labor force groups. During each of the 14 points of data collection between 1967 and 1987, women were classified as being *in the labor force* if they (1) reported that their main activity most of the last week was working, with other response categories being keeping house, going to school, looking for work, unable to work, or other; (2) worked at all last week, not including around the house; or (3) were temporarily absent from a job or business last week. The two other labor force groups include those who were actively looking for work and those who were neither working nor looking for work the previous week. Because the NLS-MW is a long-term longitudinal study and attempts were made to contact the original sample at each wave, some women dropped in and out of the study, and the amount of available information thus varies by person. However, among the 3,353 women eligible for our sample, nearly 84% had complete nonmissing labor force information for all 14 waves, with just 1% of women missing information on more than three of the waves. We drop one respondent who was missing more than half the labor force status data from the analyses. We measure work histories with the *Proportion of waves worked, 1967–1987*, allowing us to compare women with unequal rates of participation in the study by dividing the number of times women reported being in the labor force by the number of waves they were not missing information on this variable.^3^ This interval measure of women’s midlife work ranges from 0 to 1. Based on this summary variable, 13.1% of women never worked outside the home during this period, 27.7% of women worked at least one-half of the times they were interviewed, and 59.2% were working more than one-half of the waves they were interviewed.

To help account for the possibility that midlife work histories will influence late-life circumstances indirectly through their effects on more proximate work statuses (e.g., Halpern-Manners et al. 2015; Raymo et al. 2013) as well as to assess whether any long-term health effects of midlife work persist after considering more recent employment status (Moore and Hayward 1990), we also include a binary variable for *Worked last valid interview, 1989–2003* in these analyses. Women were coded 1 if they reported that work was their main activity during the previous week the last time that they were interviewed.

Last, we include *Age at first job* to account for heterogeneity in when women’s working lives began. Women who were working or had worked at first interview reported the year their first or current job began. For these women, the ages when they first worked ranged from 5 to 44. Women who reported working for the first time in subsequent waves up to 1987 also were assigned a value, increasing the age range to 58 years. By 1987, 57 women had never worked, and they received a score of 0 on this measure.

#### *Work Characteristics, 1967–1987*

We also assess the effects of a number of structural characteristics of women’s work histories as well as subjective aspects and perceptions of their work experiences during midlife. Women who never worked from 1967 to 1987 receive a score of 0 on each of the following measures.

##### Main occupation group

We create three dummy variables for respondents’ most frequently reported employment group based on data from all 14 waves of the NLS-MW from 1967 to 1987. *Blue collar* identifies women who most frequently worked in the farming or industrial/operative industries or were laborers. *Pink collar* includes women who most often reported working in sales, household service, and other service industries during this period. *White collar* workers include those who most often reported working in managerial or other professional jobs as well as women who were employed by the military. Women who reported being in different occupation groups the same number of times during these 14 waves (*N* = 148) were assigned to the higher occupation group.

##### Average hours worked

All women in the labor force were asked how many hours they worked the previous week. To create a summary variable of this measure that adjusts for varying response rates, we calculate the average number of hours women reported working during weeks that they worked. We drop one respondent who only had one wave of nonmissing data on work hours from the analysis. Scores on this variable range from 0 to 107.

##### Proportion of waves reported work discrimination

At four points from 1967 to 1987 (1972, 1977, 1982, and 1987), women were asked, “In the past 5 years do you feel that, so far as work is concerned, you have been in any way discriminated against because of race, religion, sex, age, marital status, nationality, disability, or for any other reason?” In 1972 and 1977, only women who reported working in the last five years were asked about these experiences; in 1982 and 1987, all women were asked regardless of their labor force participation. We divide the number of waves that working women perceived work-related discrimination by the number of responses given to create the proportion variable, ranging from 0 = never reported work discrimination to 1 = reported work discrimination every wave.

##### Average negative job attitude score

During 10 of the 14 waves from 1967–1987 (1967, 1971, 1972, 1977, 1979, 1981, 1982, 1984, 1986, and 1987), women who were in the labor force were asked, “How do you feel about the job you have now?” The four response categories range from “Dislike it very much” (coded 3) to “Like it very much” (coded 0). We sum the scores on this question across all 10 waves and divide by the number of waves that women had nonmissing data on this question to create an average score for negative work attitudes during this period, which ranges from 0 to 3.

##### Proportion of waves reported lack of job commitment

The NLS-MW also measured working women’s commitment to work at 10 points during this period (1967, 1972, 1974, 1976, 1977, 1981, 1982, 1984, 1986, and 1987). Respondents were asked, “If, by some chance you were to get enough money to live comfortably without working, do you think that you would work anyway?” Response categories were “Yes,” “Undecided,” or “No.” We divide the number of times women responded “no” to this question by the number of these waves they were present to create a proportion that facilitates the comparison of women with different survey participation rates.

#### *Other Sociodemographic and Control Variables*

We include controls for several other sociodemographic characteristics that may explain the relationship between employment histories and long-term health. These include respondents’ *Race* (African American compared with White); *Educational attainment* (0–6 years of schooling, 7–11 years, and 12 years, compared with 13 or more years); and *Age*, *Marital status*, *Parental status*, and *Logged family income* at the end of the work history focus period in 1987.^4^ We also include two measures of women’s health at baseline in 1967 to help account for the selection of healthier women into work. First is a dichotomous control for *Health limitation* based on a question asking whether they had a health problem that limited the type or amount of work or housework they could do. Those responding “yes” are scored as 1 on this measure. Second is a measure of *Self-rated health*, which indicates whether women rated their health as poor, fair, good, or excellent compared with other women of their age. This item ranges from 0 (poor) to 3 (excellent).

### Analytic Approach

We use linear growth curve models to estimate changes in women’s depressive symptoms and functional limitations from 1989 to 2003. These nested analyses allow for multiple within-person observations and thus provide information about intraindividual changes in physical and mental health over time as well as estimates of the relationships between independent variables (specifically, work histories from 1967 to 1987) and these outcomes over time. Time is coded as 0 at the first measurement period in 1989. Based on likelihood ratio tests, the inclusion of time as a random effect produces a better-fitting model than without. As described earlier, we include respondents who had information on the dependent variable from at least two time points in the analyses for each outcome. To explore differences in age-specific mortality based on work histories, we employ Cox proportional hazards regression (Cox 1972). These analyses estimate how hazard rates of death from age 49 to 90 are associated with predictor variables, under the assumption that these risks are proportional to the baseline hazard rate. We test the proportional hazards assumption by including interactions between age and work histories, which are not significant predictors of mortality.

Although we employ two types of longitudinal models to fit the different outcomes, the basic sequence of models is similar. We begin with models including controls for age, health at baseline in 1967, race, education, marital status, and parental status in addition to the proportional measure indicating waves worked from 1967 to 1987. These analyses show whether women’s frequency of reported work over these 20 midlife years is related to their long-term depressive symptoms, functional limitations, and mortality after we adjust for other key demographic predictors of both work and health. Next, we add a control for the age when women first worked for pay to account for heterogeneity in when women’s working lives began as well as one identifying those who reported working during their last valid interview from 1989 to 2003 to examine whether health effects of earlier work persistence emerge primarily through their effects on later work status. We then add structural factors related to work that may impact health, including logged family income, occupation group, and hours worked. Next we include women’s negative experiences with work from 1967 to 1987 (work discrimination, negative attitude toward job, and job commitment) to explore whether they help explain any health benefits of work. Finally, we add three terms interacting women’s midlife subjective work experiences and consistency to assess whether the relationship between work and the three outcomes differs for women with positive and negative experiences. We also include interactions between work histories and time in models for depressive symptoms and functional limitations to assess whether the relationship between these experiences and later health diminishes, persists, or increases over time.

## Results

### Bivariate Analysis

Table 2 shows frequencies and means of study variables as well as differences in these variables by work history from 1967 to 1987. For the purposes of providing descriptive information about differences among women who worked more and less frequently during these two decades, we divide the sample into those who never worked during this period, those who worked less than one-half of the waves interviewed, and those who worked more than one-half the time. Hereafter, these groups are referred to a nonworkers, inconsistent workers, and consistent workers, respectively.

**Table 2:** Descriptive statistics for study variables by work history, 1967–1987

	All (*N* = 3,207)	Not in Labor Force, 1967–1987 (*N* = 421)	Worked <50% Waves, 1967–1987 (*N* = 889)	Worked 50% + Waves, 1967–1987 (*N* = 1,897)
Died by 2012 (%)	40.04	47.74	43.31	36.79*^
Depressive Symptoms, 1989 (0–21)^a^	3.75 (4.35)	4.41 (4.70)	4.39 (4.85)	3.51*^ (4.13)
Functional Limitations, 1989 (0–5)^a^	0.95 (1.38)	1.28 (1.51)	1.26 (1.53)	0.74*^ (1.23)
Health Prevented or Limited Work, 1967 (%)	16.84	28.50	21.15*	12.23*^
Self-rated Health, 1967 (0–3)	2.25	1.95	2.15*	2.37*^
	(0.78)	(0.94)	(0.81)	(0.69)
Age, 1987 (50–64)	57.08	58.22	57.11*	56.81*
	(4.36)	(4.38)	(4.43)	(4.27)
African American (%)	26.69	24.47	26.10	27.46
Education (%)				
0–6 years of education	6.80	14.96	7.99*	4.43*^
7–11 years of education	34.14	36.82	39.37	31.10*^
12 years of education	41.97	37.77	37.91	44.81*^
13+ years of education	17.09	10.45	14.74*	19.66*^
Marital Status, 1987 (%)				
Married	69.25	76.46	75.14	65.10*^
Divorced	10.79	4.28	7.20*	13.92*^
Widowed	15.75	15.68	16.20	15.55
Never married	4.12	3.80	1.46*	5.43^
Has Living Children, 1986 (%)	90.33	92.87	93.03	88.51*^
Worked Last Valid Interview, 1989–2003 (%)	11.47	2.14	8.55*	14.92*^
Natural Log of Family Income, 1987	11.73 (0.15)	11.71 (0.17)	11.71 (0.15)	11.75*^ (0.15)
Age at First Job (0–58)			23.09	22.39^
			(9.52)	(7.80)
Main Occupation Group, 1967–1987 (%)				
Blue collar			17.44	17.29
Pink collar			44.54	26.94^
White collar			36.33	55.77^
Average Hours Worked, 1967–1987 (0–107)	––	––	28.78 (13.97)	35.53^ (8.39)
Proportion Waves Reported Discrimination, 1967–1987	––	––	.08 (.17)	.14^ (.24)
Average Negative Job Attitude Score 1967–1987 (0–3)	––	––	0.49 (0.55)	0.47 (0.40)
Proportion Waves Expressed Lack of Job Commitment 1967–1987	––	––	.39 (.36)	.35^ (.30)

*Note:* Standard deviations are shown in parentheses.

^a^*N* = 2,781.

*Significantly different from those who never worked, 1967–1987, *p* < .05.

^Significantly different from those who worked less than half waves, 1967–1987, *p* < .05.

Just over 40% of the sample had died by 2012, and mortality rates were significantly lower among consistent workers than nonworkers and inconsistent workers. Depressive symptoms and functional limitations in 1989 were also lower among consistent workers, providing bivariate support for the hypothesis that consistent midlife work predicts better health and mortality in later life. Both groups of women who worked were less likely to have a health limitation and rated their health higher at baseline than nonworkers. Also, consistent workers were less likely to have limitations and reported better self-rated health than inconsistent workers.

Table 2 shows additional sociodemographic differences between these groups. Women who worked during 1967–1987 were, on average, somewhat younger and better educated than nonworkers. Consistent workers were also younger and had more years of education than inconsistent workers. Whereas both groups of midlife workers were more likely to be divorced than nonworkers in 1987, consistent workers were less likely to be married than both groups. Inconsistent workers had the lowest odds of having never been married in 1987. As anticipated, women who worked—particularly those who worked consistently—were more likely to have reported paid work when last interviewed. Consistent workers also had higher family incomes in 1987.

Among workers, consistent workers were more often employed in white-collar jobs, and inconsistent workers were more likely to work in pink-collar fields. Women who worked consistently also entered the labor force at slightly younger ages and worked more hours on average. Consistent workers were more likely to report experiencing discrimination at work and to be committed to work.^5^ In short, Table 2 shows that when bivariate relationships are considered, women who worked (and worked more consistently) from 1967 to 1987 had better health earlier and later in life as well as greater socioeconomic resources than nonworkers, and reported relatively positive experiences on the job.

### Depressive Symptoms

Moving onto the first set of multivariate analyses, Table 3 shows fixed effects and covariance parameters for linear growth models regressing repeated measures of depressive symptoms from 1989 to 2003 on work histories from 1967 to 1987. Model 1 shows that women’s depressive symptoms scores increased somewhat over time and that the proportion of waves worked from 1967 to 1987 is a negative predictor of depressive symptoms at *p* < .001. In other words, consistent workers had depressive symptoms scores that were nearly one point (0.919) lower on average than women who never worked. This is after accounting for several other significant sociodemographic predictors of work histories and depressive symptoms: those with health limitations at baseline and African American women reported higher depressive symptoms scores, while depressive symptoms decreased as baseline self-rated health and age increased. Likelihood ratio tests indicate that the random effect for time (or the rate of change) of 0.195 is significant, which means that the change in depressive symptoms over time varied by person.

**Table 3:** Fixed effects and covariance parameters for growth models regressing depressive symptoms in 1989, 1995, 1997, 1999, 2001, and 2003 on work history: 1967–1987 (*N* = 2,781)

	Model 1	Model 2	Model 3	Model 4	Model 5	Model 6
Time	0.045*	0.078*	0.078*	0.079*	0.078*	0.078*
	(0.018)	(0.034)	(0.034)	(0.034)	(0.034)	(0.034)
Proportion of Waves Worked, 1967–1987	–0.919*** (0.175)	–0.799*** (0.205)	–0.744*** (0.206)	–0.722** (0.248)	–0.882** (0.246)	–0.869** (0.331)
Age, 1987	0.020	0.021	0.007	0.001	0.002	0.003
	(0.013)	(0.013)	(0.013)	(0.013)	(0.013)	(0.013)
Health Prevented/Limited Type/Amount of Work, 1967	1.068*** (0.175)	1.068*** (0.175)	1.072*** (0.175)	1.024*** (0.174)	1.015*** (0.172)	1.016*** (0.172)
Self-rated Health, 1967	–0.657***	–0.657***	–0.661***	–0.631***	–0.611***	–0.607***
	(0.089)	(0.089)	(0.088)	(0.088)	(0.087)	(0.087)
African American	0.450**	0.450**	0.384**	0.207	0.225	0.226
	(0.143)	(0.143)	(0.143)	(0.148)	(0.146)	(0.146)
Years of Education (ref. = 13+ years)						
0–6 years	2.172***	2.173***	2.170***	1.767***	1.805***	1.803**
	(0.275)	(0.275)	(0.274)	(0.287)	(0.285)	(0.285)
7–11 years	1.483***	1.483***	1.489***	1.125***	1.110***	1.092***
	(0.177)	(0.177)	(0.176)	(0.193)	(0.191)	(0.192)
12 years	0.461**	0.461**	0.492**	0.303	0.278	0.273
	(0.159)	(0.159)	(0.160)	(0.163)	(0.162)	(0.163)
Marital Status, 1987 (ref. = never married)						
Married	–0.146	–0.145	–0.060	0.118	0.096	0.089
	(0.321)	(0.321)	(0.321)	(0.323)	(0.319)	(0.319)
Divorced	0.131	0.133	0.280	0.254	0.176	0.169
	(0.354)	(0.354)	(0.354)	(0.354)	(0.350)	(0.350)
Widowed	–0.046	–0.045	0.058	0.081	0.088	0.080
	(0.343)	(0.343)	(0.343)	(0.342)	(0.338)	(0.338)
Has Living Children, 1986	0.146 (0.215)	0.148 (0.215)	0.145 (0.214)	0.181 (0.214)	0.122 (0.212)	0.120 (0.212)
Proportion of Waves Worked, 1967–1987 × Time		–0.058 (0.052)	–0.058 (0.052)	–0.059 (0.052)	–0.057 (0.052)	–0.058 (0.052)
Worked Last Valid Interview, 1989–2003			–0.818*** (0.189)	–0.815*** (0.188)	–0.789*** (0.187)	–0.804*** (0.187)
Age at First Job			0.015*	0.011	0.010	0.009
			(0.007)	(0.007)	(0.007)	(0.007)
Natural Log of Family Income, 1987				–2.084*** (0.435)	–1.879*** (0.431)	–1.828*** (0.432)
Main Occupation Group, 1967–1987 (ref. = blue collar)						
White collar				–0.113	–0.222	–0.233
				(0.171)	(0.171)	(0.175)
Pink collar				0.227	0.134	0.121
				(0.162)	(0.161)	(0.166)
Average Hours Worked, 1967–1987				0.005 (0.005)	–0.003 (0.005)	–0.003 (0.005)
Proportion of Waves With Reported Discrimination, 1967–1987					1.326*** (0.275)	2.516** (0.777)
Average Negative Job Attitude Score, 1967–1987					0.362** (0.132)	0.027 (0.230)
Proportion of Waves With Reported Lack of Job Commitment 1967–1987					0.771*** (0.188)	1.029** (0.359)
Proportion of Waves Worked, 1967–1987 × Proportion of Waves With Reported Discrimination, 1967–1987						–0.704 (1.005)
Proportion of Waves Worked, 1967–1987 × Average Negative Job Attitude Score, 1967–1987						0.689 (0.400)
Proportion of Waves Worked, 1967–1987 × Proportion of Waves With Reported Lack of Job Commitment, 1967–1987						–0.528 (0.572)
Random Effect						
Intercept variance	7.270	7.268	7.234	7.165	6.951	6.926
Rate of change	.195	.195	.196	.196	.196	.196
Residual variance	8.878	8.878	8.878	8.875	8.874	8.874
Log-Likelihood	–37,467.483	–37,466.853	–37,454.867	–37,438.793	–37,408.477	–37,405.818

*Note:* Standard errors are shown in parentheses.

*p < .05; **p < .01; ***p < .001

In Model 2, we add an interaction term for the proportion of waves worked and time, which is not significant. This suggests that rather than growing or diminishing, the relationship between depressive symptoms and these work histories remained stable over the following 14 years. Model 3 includes the dichotomous measure that indicates whether women worked during the last wave for which we had valid information. Although this proximate work status is a highly significant predictor of decreased depressive symptoms, it does not explain the relationship between consistent work and later mental health. Among those who worked, depressive symptoms scores were somewhat lower for women who started working at a relatively young age and increased along with age at first job.

Model 4 adds several variables that are correlated with structural aspects of women’s jobs that may also carry long-term health benefits. Logged family income in 1987 was negatively associated with depressive symptom scores, although neither most frequent occupation group nor average hours worked from 1967 to 1987 were related to depressive symptoms. Further, the proportion of waves worked during midlife remains a significant negative predictor of later-life depressive symptoms. In Model 5, we add three variables averaging work experiences and appraisals from 1967 to 1987: perceptions of discrimination, negative attitude toward job, and lack of commitment to work. Women who consistently reported work discrimination acknowledged more depressive symptoms than nonworking women or those who did not perceive discriminatory behaviors at *p* < .001, making it almost as strong of a predictor of later depression as educational attainment. Women who often expressed being uncommitted to continuing work also had higher depressive symptoms scores. Finally, Model 6 incorporates interaction terms for each subjective work experience and midlife work consistency. Because these work characteristics affect only women who worked—not the nonworking portion of the sample—they act as internal moderators (Mirowsky 2013). The coefficient for the proportion of waves worked in this model is thus interpretable as the effect of work for women with scores of 0 on each of the subjective work experiences: in other words, the women who had the most positive average experiences at work in midlife. These interaction terms are not significant, suggesting that the relationship between work and depressive symptoms did not differ between women with the best work experiences and women with the most negative work experiences. We also explore the effects of interactions between time and each of the measures of midlife work characteristics, shown in Table A6 in the online appendix. None of these terms was significant, indicating stability in the relationships between work characteristics and later depression.

### Functional Limitations

Analyses in Table 4 are parallel to those in Table 3 but with functional limitations as the outcome of interest. Intuitively—given that physical health problems increase with age—Model 1 first shows that functional limitations increased as the study progressed from 1989 to 2003. As with depressive symptoms, this model next shows a protective effect of midlife work consistence; women who worked stably had functional limitations scores that were 0.484 lower than their nonworking counterparts of the same age, race, education, and marital and parental status. Model 1 also shows that functional limitations increased with age and were higher, on average, for women who reported a health limitation at baseline, had worse self-rated health, were African American, and had less than a high school education. The random effect for time (0.044) is again significant, indicating that time effects varied across individuals and supporting the use of these nested models over linear approaches.

**Table 4:** Fixed effects and covariance parameters for growth models regressing functional limitations in 1989, 1995, 1997, 1999, 2001, and 2003 on work history: 1967–1987 (*N* = 2,781)

	Model 1	Model 2	Model 3	Model 4	Model 5	Model 6
Time	0.128***	0.129***	0.128***	0.128***	0.128***	0.128***
	(0.007)	(0.013)	(0.013)	(0.013)	(0.013)	(0.013)
Proportion of Waves Worked, 1967–1987	–0.484*** (0.062)	–0.483*** (0.067)	–0.449*** (0.068)	–0.535*** (0.083)	–0.575*** (0.083)	–0.554*** (0.114)
Age, 1987	0.030***	0.030***	0.024***	0.022***	0.022***	0.022***
	(0.005)	(0.005)	(0.005)	(0.005)	(0.005)	(0.005)
Health Prevented/Limited Type/Amount of Work, 1967	0.506*** (0.062)	0.506*** (0.062)	0.505*** (0.062)	0.489*** (0.061)	0.480*** (0.061)	0.480*** (0.061)
Self-rated Health, 1967	–0.226*** (0.031)	–0.226*** (0.031)	–0.228*** (0.031)	–0.222*** (0.031)	–0.219*** (0.031)	–0.218*** (0.031)
African American	0.245***	0.245***	0.215***	0.182***	0.189***	0.189***
	(0.051)	(0.051)	(0.051)	(0.052)	(0.052)	(0.052)
Years of Education (ref. = 13+ years)						
0–6 years of education	0.650*** (0.098)	0.650*** (0.098)	0.651*** (0.097)	0.574*** (0.102)	0.591*** (0.101)	0.590*** (0.101)
7–11 years of education	0.437*** (0.063)	0.437*** (0.063)	0.441*** (0.062)	0.367*** (0.068)	0.370*** (0.068)	0.367*** (0.068)
12 years of education	0.112* (0.057)	0.112* (0.057)	0.123* (0.057)	0.079 (0.058)	0.080 (0.058)	0.078 (0.058)
Marital Status, 1987 (ref. = never married)						
Married	–0.117	–0.117	–0.084	–0.035	–0.043	–0.045
	(0.114)	(0.114)	(0.113)	(0.114)	(0.113)	(0.113)
Divorced	0.115	0.115	0.182	0.155	0.127	0.126
	(0.125)	(0.125)	(0.125)	(0.125)	(0.124)	(0.120)
Widowed	–0.082	–0.082	–0.040	–0.038	–0.042	–0.045
	(0.121)	(0.121)	(0.121)	(0.121)	(0.120)	(0.120)
Has Living Children, 1986	0.020 (0.076)	0.020 (0.076)	0.018 (0.076)	0.044 (0.076)	0.031 (0.075)	0.029 (0.075)
Proportion of Waves Worked, 1967–1987 × Time		–0.001 (0.019)	–0.001 (0.019)	–0.001 (0.019)	–0.000 (0.019)	–0.000 (0.019)
Worked Last Valid Interview, 1989–2003			–0.437*** (0.067)	–0.439*** (0.067)	–0.436*** (0.066)	–0.438*** (0.066)
Age at First Job			0.006*	0.004	0.004	0.004
			(0.002)	(0.002)	(0.002)	(0.002)
Natural Log of Family Income, 1987				–0.680*** (0.154)	–0.640*** (0.153)	–0.627*** (0.154)
Main Occupation Group, 1967–1987 (ref. = blue collar)						
White collar				0.059	0.014	0.008
				(0.060)	(0.060)	(0.062)
Pink collar				0.063	0.036	0.031
				(0.057)	(0.057)	(0.059)
Average Hours Worked, 1967–1987				0.004* (0.002)	0.002 (0.002)	0.002 (0.002)
Proportion of Waves With Reported Work Discrimination, 1967–1987					0.464*** (0.098)	0.759** (0.262)
Average Negative Job Attitude Score, 1967–1987					–0.024 (0.447)	–0.071 (0.082)
Proportion of Waves With Reported Lack of Job Commitment, 1967–1987					0.257*** (0.066)	0.297* (0.127)
Proportion of Waves Worked, 1967–1987 × Proportion of Waves With Reported Discrimination, 1967–1987						–0.414 (0.356)
Proportion of Waves Worked, 1967–1987 × Average Negative Job Attitude Score, 1967–1987						0.095 (0.142)
Proportion of Waves Worked, 1967–1987 × Proportion of Waves With Reported Lack of Job Commitment, 1967–1987						–0.083 (0.202)
Random Effect						
Intercept variance	.774	.775	.767	.764	.746	.746
Rate of change	.044	.044	.044	.044	.044	.044
Residual variance	.944	.944	.944	.944	.944	.944
Log-Likelihood	–22,820.207	–22,795.959	–22,795.959	–22,782.558	–22,763.722	–22,762.889

*Note:* Standard errors are shown in parentheses.

*p < .05; **p < .01; ***p < .001

To assess whether the effects of work consistency in midlife changed from 1989 to 2003, Model 2 includes an interaction between work history and time. Consistent with results for depressive symptoms, the coefficient is again nonsignificant, suggesting that by 1989, these relationships were stable. Model 3 shows that although women who worked during their most recent completed interview had lower functional limitations scores, the effect of this proximate work measure does not explain the relationship between midlife work history and later functional health, nor does accounting for the age at which they first began working, which is modestly positively associated with functional limitations. Two measures correlated with the structure of women’s midlife work in 1989 also predict functional limitations in Model 4: as income from 1967 to 1987 increased, functional limitations later in life decreased, and limitations increased somewhat with hours worked. Model 5 shows the strong negative health effects of experiences with work discrimination and lack of work commitment, which are comparable with the depressing effects of having health limitations at baseline and being low education. Again, the strong relationship between work consistency during the first 20 years of the study and functional limitations over 14 years later persists. Last, Model 6 adds interaction terms for subjective work experiences and midlife work consistency. They are again not significant, indicating that these experiences did not moderate the relationship between midlife work and later-life functional limitations. Supplemental analyses exploring whether the associations between work characteristics and experiences are conditional on time also yielded nonsignificant results with the exception of the term for white collar (see Table A6, online appendix). This interaction term is negative and significant at *p* < .01, suggesting that functional limitations increased at a slower rate for women who were in white-collar compared with blue-collar jobs.

### Mortality

Analyses in Table 5 predict mortality rates using Cox proportional hazards models. Age at death is the dependent variable: women who died between 1989 and 2012 have an observed age of death, and women who survived are right-censored. Addressing our central question about the relationship between work consistency and longevity, Model 1 indicates that consistent workers survived to older ages than others. More specifically, women who worked during all their interviews had a 25.1% lower risk of dying over the 23-year follow-up period. Model 1 also shows that women who were older in 1987, were African American, or had less than 13 years of education had greater risks of death than younger, White, and more-educated women. In addition, the risk of death decreased as self-rated health at baseline increased. Model 2 adds the measure of most recent reported work status, which is also associated with decreased mortality risk at *p* < .05 but does not reduce the effect of earlier work consistency. The age at which women first entered work is not related to their risk of dying over this period. After adding characteristics of work in Model 4, income in 1987 was negatively associated with the mortality hazard, but the effect of women’s first 20 years of work consistency remains strong. None of the measures of work experiences in Model 5 significantly predict mortality. Interactions between subjective work experiences and midlife work consistency included in Model 6 are also not significant, suggesting that the long-term longevity advantage of consistently working women is not dependent on these experiences.

**Table 5:** Hazard ratios from Cox proportional hazards models of women’s age-dependent mortality to 2012 by work history, 1967–1987 (*N* = 3,207)

	Model 1	Model 2	Model 3	Model 4	Model 5
Proportion of Waves Worked, 1967–1987	0.0749***	0.0773**	0.0794*	0.0794*	0.0724*
	(0.0061)	(0.0063)	(0.0084)	(0.0085)	(0.0115)
Age, 1987	1.054***	1.051***	1.049***	1.049***	1.048***
	(0.0007)	(0.0007)	(0.0007)	(0.0007)	(0.0007)
Health Prevented/Limited Type/Amount of Work, 1967	1.017 (0.0079)	1.021 (0.0079)	1.009 (0.0078)	1.007 (0.0078)	1.002 (0.0078)
Self-rated Health, 1967	0.0866***	0.0866***	0.0871***	0.0871**	0.0871**
	(0.0036)	(0.0036)	(0.0036)	(0.0036)	(0.0036)
African American	1.364***	1.359***	1.287***	1.289***	1.288***
	(0.0088)	(0.0088)	(0.0086)	(0.0086)	(0.0086)
Years of Education (ref. = 13+ years)					
0–6 years	1.404**	1.395**	1.248	1.267	1.254
	(0.0180)	(0.0179)	(0.0167)	(0.0170)	(0.0169)
7–11 years	1.536***	1.541***	1.397***	1.413***	1.404***
	(0.0142)	(0.0143)	(0.0139)	(0.0141)	(0.0140)
12 years	1.255*	1.245*	1.181	1.189	1.179
	(0.0113)	(0.0113)	(0.0109)	(0.0110)	(0.0109)
Marital Status, 1987 (ref. = never married)					
Married	0.0853	0.0847	0.0893	0.0896	0.0893
	(0.0118)	(0.0118)	(0.0125)	(0.0126)	(0.0126)
Divorced	1.020	1.031	1.030	1.020	1.019
	(0.0158)	(0.0161)	(0.0161)	(0.0160)	(0.0161)
Widowed	0.0891	0.0890	0.0897	0.0897	0.0893
	(0.0130)	(0.0131)	(0.0132)	(0.0132)	(0.0132)
Has Living Children, 1986	0.0882	0.0884	0.0883	0.0882	0.0880
	(0.0079)	(0.0080)	(0.0080)	(0.0080)	(0.0080)
Worked Last Valid Interview, 1989–2003		0.0787*	0.0793*	0.0789*	0.0798*
		(0.0087)	(0.0088)	(0.0088)	(0.0089)
Age at First Job		0.0998	0.0997	0.0997	0.0997
		(0.0003)	(0.0003)	(0.0003)	(0.0003)
Natural Log of Family Income, 1987			0.0489**	0.0489**	0.0486**
			(0.0114)	(0.0114)	(0.0114)
Main Occupation Group, 1967–1987 (ref. = blue collar)					
White collar			1.002	0.0982	0.0983
			(0.0085)	(0.0088)	(0.0086)
Pink collar			1.099	1.099	1.104
			(0.0081)	(0.0082)	(0.0084)
Average Hours Worked, 1967–1987			1.000	0.0999	0.0999
			(0.0002)	(0.0002)	(0.0002)
Proportion of Waves With Reported Discrimination, 1967–1987				1.278 (0.0179)	1.148 (0.0440)
Average Negative Job Attitude Score, 1967–1987				0.0912 (0.0058)	0.0982 (0.0103)
Proportion of Waves With Reported Lack of Job Commitment, 1967–1987				1.073 (0.0050)	0.0925 (0.0114)
Proportion of Waves Worked, 1967–1987 × Proportion of Waves With Reported Discrimination, 1967–1987					1.187 (0.0603)
Proportion of Waves Worked, 1967–1987 × Average Negative Job Attitude Score, 1967–1987					0.0832 (0.0161)
Proportion of Waves Worked, 1967–1987 × Proportion of Waves With Reported Lack of Job Commitment, 1967–1987					1.456 (0.0336)

*Note:* Standard errors are shown in parentheses.

*p < .05; **p < .01; ***p < .001

### Sensitivity Analyses

We further explored issues with the causal patterning of the work-health relationship by creating a variable that summarizes health limitations reported from 1967 to 1976, and assessing how work from the period 1977–1987 was related to later health and mortality while controlling for this longer-term baseline measure of early health. The results, summarized in Table 6, are consistent with those in Tables 3, 4, and 5: even accounting for prior health limitations over a period of 9 years, consistent work over the following 10 years predicted fewer depressive symptoms and functional limitations and lower risk of death over the following 16+ years.

**Table 6:** Results from regressions of depressive symptoms, functional limitations, and mortality on work history, 1977–1987

	Depressive Symptoms (*N* = 2,781)	Functional Limitations (*N* = 2,781)	Mortality (HR) (*N* = 3,207)
Proportion of Waves Worked, 1977–1987	–0.488*	–0.387***	0.770*
	(0.246)	(0.082)	(0.088)
Main Occupation Group, 1977–1987 (ref. = blue collar)			
White collar	–0.304	–0.025	0.998
	(0.168)	(0.059)	(0.003)
Pink collar	0.081	0.011	1.019
	(0.157)	(0.056)	(0.067)
Average Hours Worked, 1977–1987	–0.002	0.002	1.000
	(0.005)	(0.002)	(0.002)
Proportion of Waves With Reported Discrimination. 1977–1987	1.133***	0.371***	1.052
	(0.271)	(0.095)	(0.118)
Average Negative Job Attitude Score, 1977–1987	0.344***	–0.031	1.001
	(0.129)	(0.045)	(0.039)
Proportion of Waves With Reported Lack of Job Commitment, 1977–1987	0.798*** (0.184)	0.268*** (0.065)	0.973 (0.069)
Average Reports of Health Limitations, 1967–1976	2.764***	1.335***	1.417***
	(0.278)	(0.097)	(0.138)

*Notes:* All models include age, baseline health, race, education, marital status in 1987, children in 1986, interactions for time and waves worked (for depressive symptoms and functional limitations), last valid work status, age at first job, and income in 1987. Standard errors are shown in parentheses.

*p < .05; ***p < .001

Finally, acknowledging meaningful racial patterning in work experiences (e.g., Hayward et al. 1996), we assessed whether there were differences in the relationship between White and African American women’s work experiences and their later health and mortality with interaction terms (see Table 7). However, none of these interactions reached significance, indicating that the relationship between midlife work and later health and mortality was similar for White and African American women in this cohort.

**Table 7:** Results from regressions of depressive symptoms, functional limitations, and mortality on work history from 1967–1987 with interactions with race

	Depressive Symptoms (*N* = 2,781)	Functional Limitations (*N* = 2,781)	Mortality (HR) (*N* = 3,207)
Proportion of Waves Worked, 1967–1987	–0.804**	–0.511***	0.850
	(0.282)	(0.096)	(0.115)
African American	0.606	0.375**	1.242
	(0.340)	(0.120)	(0.185)
Proportion of Waves Worked, 1967–1987 × African American	–0.348 (0.478)	–0.205 (0.170)	0.833 (0.182)
Main Occupation Group, 1967–1987 (ref. = blue collar)			
White collar	–0.309	–0.004	0.937
	(0.192)	(0.111)	(0.091)
Pink collar	–0.117	–0.030	1.031
	(0.202)	(0.071)	(0.099)
White Collar × African American	–0.040	–0.099	1.120
	(0.392)	(0.139)	(0.219)
Pink Collar × African American	0.610	0.165	1.185
	(0.335)	(0.119)	(0.184)
Average Hours Worked, 1967–1987	0.001	0.003	1.001
	(0.006)	(0.002)	(0.003)
Average Hours Worked, 1967–1987 × African American	–0.014	–0.004	0.997
	(0.011)	(0.004)	(0.005)
Proportion of Waves With Reported Discrimination, 1967–1987	1.251*** (0.315)	0.378** (0.227)	1.210 (0.203)
Discrimination, 1967–1987 × African American	0.322	0.329	1.143
	(0.640)	(0.227)	(0.350)
Average Negative Job Attitude Score, 1967–1987	0.478**	–0.055	0.862
	(0.156)	(0.055)	(0.070)
Average Negative Job Attitude Score, 1967–1987 × African American	–0.359 (0.291)	0.096 (0.103)	1.160 (0.153)
Proportion of Waves With Lack of Job Commitment, 1967–1987	0.626*** (0.217)	0.299*** (0.077)	1.040 (0.101)
Lack of Job Commitment 1967–1987 × African American	0.512 (0.428)	–0.162 (0.152)	1.126 (0.193)

*Notes:* All models include age, baseline health, education, marital status in 1987, children in 1986, interactions for time and waves worked (for depressive symptoms and functional limitations), last valid work status, age at first job, and income in 1987. Standard errors are shown in parentheses.

**p < .01; ***p < .001

## Discussion

Although it is now widely accepted that paid work has significant health benefits for women as well as men, a number of questions about how this relationship plays out over the course of adults’ lives remain. Our analysis draws on decades of information about women’s midlife work experiences as well as their later-life health and mortality to address three specific gaps in research about the longitudinal relationship between work and health. Our first goal was to elaborate knowledge about whether the relationship between work and health persists over long periods. Second, we explored the role of subjective experiences with and appraisals of work in conditioning the health/work relationship. And third, we assessed whether the long-term benefits of work were dependent on the health indicator being examined.

Our analyses show that women who worked consistently over a 20-year period in midlife reported fewer depressive symptoms and functional limitations over the following 16 years as they entered later life. They also enjoyed longer lives over the next 25 years than their counterparts who did not work during this extended period. These findings hold even after we accounted for women’s most proximate work status, baseline health, and structural characteristics and appraisals of these jobs. Consistent with other longitudinal work studies, we also found evidence for health selection in supplemental analyses: women in better health and without limitations at baseline were more likely to work during the next two decades, and women who reported depressive symptoms or had more functional limitations during the work measurement period had higher odds of leaving the study before 1989. However, the findings remain significant when we control for baseline health and in supplemental analyses adjusting for nonrandom, health-related attrition from the sample (Table A1, online appendix). Our main analyses also assess whether health differences between women who consistently worked in midlife and those who did not diminished, were stable, or grew over time using linear growth curve models; our findings indicate that the health advantages associated with midlife work were stable as women entered late life. In all, these findings are consistent with existing research suggesting that work has a beneficial effect on health. However, there is still work to be done surrounding the question of causality. With their longitudinal focus on adults’ work lives, the NLS surveys (including the younger cohorts) provide a strong source of data for future studies aiming to further disentangle these relationships, including by looking at the patterns between early health, work, and later health over different measurement windows.

Another goal of our analysis is to investigate whether subjective aspects and appraisals of jobs—which predict health directly as well as shape the short-term health benefits of work—moderate the relationship between work and health in the long term. We find that midlife experiences with work discrimination, expressing a negative attitude toward one’s job, and low job commitment are positively associated with depressive symptoms and functional limitations. Interaction analyses reveal, however, that the relationship between midlife work and long-term health and mortality does not significantly differ based on these experiences, inconsistent with our expectations. In other words, the benefits of paid work persist even for those with negative work experiences, and those with positive work experiences do not appear to derive additional benefits from work. Although we are limited by the available data, our analyses also suggest that structural factors associated with work—including income, occupational group, and hours—also do not explain these work benefits. Future research should continue to unravel precisely what kinds of benefits and resources work provides that increase health for the rest of adults’ lives. It is likely that several characteristics of work not captured in our data—including specific on the job stressors, working conditions, and work-related social networks—help explain this relationship.

We also believe it is important to consider the historical context in interpreting these findings—namely, that during this time, many women were entering low-status jobs or fields that were traditionally dominated by men, thus encountering resistance, discrimination, and otherwise challenging work conditions (e.g., Jacobs 1989; Reskin and Roos, 1990). Subjective work experiences may have been particularly negative and stressful for this cohort of women. Although we cannot assess this possibility or the effects of historical change with our data, the study administrators included measures of the average percentage of women in different work fields in 1967 and 1969. As shown in Table A7 of the online appendix, these early indicators of the proportion of women in respondents’ fields are not predictors of their later health, nor do they explain the health effects of midlife work, suggesting that within this cohort, the gender balance of work environments was not a salient predictor of long-term well-being. We hope that future research using data with parallel cohorts of women will assess whether the health correlates of paid work and work experiences changed as it became more normative for women.

Finally, our analyses indicate that the positive relationship between midlife work and long-term well-being is consistent across all three outcomes. However, the relationship between work experiences and the three outcome measures shows some clear variations. In particular, although experiences with discrimination and commitment to work are related to both long-term depressive symptoms and functional limitations, they are not significant predictors of mortality. These findings resonate with the work of life course epidemiologists and health psychologists, highlighting that social experiences influence well-being throughout the life course in diverse ways (e.g., Cohen and Rodriguez 1995; Kuh et al. 2003; Springer 2009; Taylor et al. 1997). They also support our argument that research explorations of long-term relationships between key adult role experiences and health as lives progress would be enhanced by including outcomes that may capture more short-term and direct effects as well as those that may emerge only after long periods. Supplemental analyses including depressive symptoms in 1989 as a covariate (Table A8, online appendix) show that although these symptoms do not mediate the relationship between work and functional limitations, they do explain the lingering relationship with mortality, underscoring that these outcomes are intertwined and that midlife work may indeed increase longevity at least in part through its effect on mental health. Our findings also point to the possibility that because of the many powerful and diverse predictors of mortality, the effects of some subjective role experiences that impact long-term health are comparatively small when it comes to longevity.

One limitation of our study is that although straightforward, the proportion of waves worked is a relatively simplistic indicator of women’s work histories. The age range of the sample is smaller than in many other surveys, but time effects may be intertwined with age effects. As shown in Table A9 in the online appendix, when we limit our focus on women’s work participation from ages 44 to 50—the ages at which we have work data for all women in the sample—work consistency still predicts better physical and mental health over the following decades. However, this more limited, age-specific work is no longer a significant predictor of mortality. Although it is possible that work occurring before age 44 or after age 50 is more significant for longevity, we think it is more likely that this more limited six-year range of information simply does not capture the modest mortality effect of stable work. Our measure of work also does not provide insight about the health effects of being out of work voluntarily versus involuntarily. In further supplemental analyses, we included measures of involuntary job losses experienced before 1967, from 1969 to 1977, and from 1979 to 1987 (Table A10, online appendix), as well as the proportion of times women reported looking for a job from 1967 to 1987 (Table A11, online appendix). These analyses indicate that neither of these indicators of involuntary nonwork is related to health or mortality from 1989 onward, suggesting that the poorer health of women who do not regularly work is not contingent on whether their unemployment was voluntary. Scholars should continue to explore questions about the meanings of time, age, and the voluntariness of work status for the relationship between work and long-term health. Additionally, although we focus on women’s work experiences during midlife, our treatment of work later in life (with a dichotomous measure for work status during the respondents’ last interview) is less detailed. Other aspects of later-life work, such as the hours worked and attitudes toward work at this life stage, may further condition the effects of midlife work on later-life health. Investigating these questions about the relationships between mid- and late-life working lives and long-term health is another promising avenue for future research.

We are also limited in our ability to make inferences about the role that women’s work lives before the study started may have played in patterning their midlife work and long-term health, given that the NLS-MW did not collect complete information about work before baseline. However, we were able to explore how transitions between school and work were related to later health and mortality in supplemental analyses. These analyses (in Table A12, online appendix) show that women who began working full-time while they were still full-time students had somewhat fewer functional limitations many years later than women who had not worked full-time by the beginning of the study in 1967. However, other variations in work-school transitions are not related to later health. Additionally, supplemental analyses restricting the sample to women who had both been married and worked at some point since leaving school in 1967 (Table A13, online appendix) show that the involuntary loss of their longest job before their first marriage is not related to women’s later-life health and mortality. Along with the controls for the age when women first entered the work force in the main analyses, the supplemental analyses indicate that these specific aspects of early work experiences do not explain subsequent relationships between midlife work and well-being. Even so, they are limited measures that do little to reveal how the consistency and types of work and nonwork at the beginning of working lives shape late-life work-health patterns. Analyses of data following individuals from younger ages, such as the more recent NLS cohorts, are needed to answer these questions.

Another limitation is that measures of baseline health that parallel those used in our analyses (i.e., scales of depressive symptoms and functional limitations) were not available. Although controlling for women’s self-rated health and their reports of having a limiting health condition help account for the selection of women who were already healthier in 1967 into consistent work over the following years, additional aspects of women’s well-being may not be captured, particularly regarding their mental health. Last, our analysis is limited in its focus on women, whose work patterns and experiences continue to be distinctly different from those of men. As even longer-term and more thorough panel data on women and men’s work lives and health become available, researchers can continue to clarify how work and work experiences throughout the life course shape health as we age.

Despite the limitations, our study highlights the significance of work for women’s health within the historical context of women’s rapid growth in labor force participation during the latter twentieth century United States. It suggests that women’s long-term health may be improved by regular participation in paid work during midlife, enhancing commitment to work, and reducing experiences with on-the-job discrimination—findings that may be of interest to work organizations, policy-makers, and scholars in public health fields. More broadly, our study adds to knowledge about the processes through which adult social experiences shape health as individuals enter later life.

## Electronic supplementary material

ESM 1 (DOCX 45 kb)

## Footnotes

1 To assess whether our analyses were sensitive to inconsistencies created by the presence of a screener and different response categories in 1989, we conducted supplemental analyses excluding the 1989 measure (Table A2, online appendix). These analyses are substantively identical to those presented, albeit with a reduced sample size.

2 Analyses coding “don’t do” responses (*N* = 318 in 1995) as missing provide substantively identical results (Table A3, online appendix).

3 Analyses restricting the sample to women with nonmissing work information at each of the 14 waves from 1967 to 1987 (Table A4, appendix) produce nearly identical results.

4 Analyses using time-varying measures of marital status and income produce substantively identical results (Table A5, online appendix).

5 The relatively low average number of waves in which working women reported discriminatory treatment may be attributable to an underdeveloped national dialogue regarding workplace discrimination and harassment at this time (e.g., Fitzgerald et al. 1997).
