# Supplementary material for: Midlife Work and Women’s Long-Term Health and Mortality
Source: Demography. 2019 Dec 11;57(1):373–402. doi: 10.1007/s13524-019-00839-6 (PMC7052029; doi:10.1007/s13524-019-00839-6)
Supplement: Supplementary file 1 — (DOCX 45 kb) [file 13524_2019_839_MOESM1_ESM.docx]

| Table A1: Summary of results from regression analyses using propensity score weights that adjust for attrition | | | |  |
| --- | --- | --- | --- | --- |
|  | Depressive Symptoms  N=2686 | Functional Limitations  N=2686 | Mortality (HR)  N=3069 | |
| Time | 0.079^*^ | 0.129^***^ |  | |
|  | (0.038) | (0.013) |  | |
| Proportion waves worked 1967-1987 | -0.560^*^ | -0.473^***^ | 0.776^*^ | |
|  | (0.268) | (0.089) | (0.088) | |
| Age 1987 | -0.001 | 0.021^***^ | 1.050^***^ | |
|  | (0.014) | (0.005) | (0.007) | |
| Health prevented/limited type/amount of work 1967 | 0.979^***^ | 0.459^***^ | 1.022 | |
|  | (0.206) | (0.071) | (0.080) | |
| Self-rated health 1967 | -0.623^***^ | -0.232^***^ | 0.872^**^ | |
|  | (0.099) | (0.035) | (0.037) | |
| African-American | 0.225 | 0.182^**^ | 1.290^***^ | |
|  | (0.165) | (0.058) | (0.089) | |
| Education (Comparison: 13+ years) |  |  |  | |
| 0-6 years of education | 1.946^***^ | 0.643^***^ | 1.221 | |
|  | (0.327) | (0.107) | (0.167) | |
| 7-11 years of education | 1.190^***^ | 0.389^***^ | 1.367^**^ | |
|  | (0.184) | (0.069) | (0.139) | |
| 12 years of education | 0.341^*^ | 0.0951 | 1.154 | |
|  | (0.135) | (0.052) | (0.108) | |
| Marital status 1987 (Comparison: Never married) |  |  |  | |
| Married | 0.132 | -0.026 | 0.816 | |
|  | (0.322) | (0.128) | (0.119) | |
| Divorced | 0.154 | 0.148 | 0.950 | |
|  | (0.355) | (0.137) | (0.153) | |
| Widowed | 0.0973 | -0.029 | 0.836 | |
|  | (0.345) | (0.135) | (0.128) | |
| Has living children 1986 | 0.192 | 0.025 | 0.877 | |
|  | (0.228) | (0.083) | (0.081) | |
| Proportion waves worked 1967 to 1987 * Time | -0.052 | 0.001 |  | |
|  | (0.054) | (0.019) |  | |
| Worked last valid interview 1989-2003 | -0.793^***^ | -0.436^***^ | 0.837 | |
|  | (0.173) | (0.052) | (0.094) | |
| Age at first job | 0.011 | 0.004 | 0.997 | |
|  | (0.008) | (0.003) | (0.003) | |
| Natural log of family income 1987 | -1.899^***^ | -0.629^***^ | 0.550^*^ | |
|  | (0.475) | (0.156) | (0.131) | |
| Main occupation group 1987-1967 (Comparison: Blue collar) |  |  |  | |
| White collar | 0.017 | 0.076 | 0.963 | |
|  | (0.183) | (0.067) | (0.087) | |
| Pink collar | 0.457^*^ | 0.101 | 1.094 | |
|  | (0.188) | (0.067) | (0.088) | |
| Average hours worked 1967-1987 | 0.008 | 0.004^*^ | 1.211 | |
|  | (0.006) | (0.002) | (0.173) | |
| Proportion waves reported discrimination 1967-1987 | 1.391^***^ | 0.439^***^ | 1.000 | |
|  | (0.276) | (0.104) | (0.003) | |
| Average positive job attitude score 1967-1987 | -0.173 | -0.026 | 1.016 | |
|  | (0.107) | (0.037) | (0.046) | |
| Proportion waves reported job commitment 1967-1987 | -0.624^**^ | -0.259^***^ | 0.890 | |
|  | (0.200) | (0.069) | (0.085) | |
| *=p<.05, **=p<.01, ***=p<.001  Note: Standard errors in parentheses. | | | |  |

| Table A2: Summary of results from regression analyses for functional limitations from 1995-2003 (N=2,613) | |  |
| --- | --- | --- |
| Proportion waves worked 1967-1987 | -0.392^***^ | |
|  | (0.107) | |
| Main occupation group 1987-1967 (Comparison: Blue collar) |  | |
| White collar | 0.039 | |
|  | (0.069) | |
| Pink collar | 0.005 | |
|  | (0.065) | |
| Average hours worked 1967-1987 | 0.002 | |
|  | (0.002) | |
| Proportion waves reported work discrimination 1967-1987 | 0.439^***^ | |
|  | (0.111) | |
| Average negative job attitude score 1967-1987 | -0.078 | |
|  | (0.053) | |
| Proportion waves reported lack of job commitment 1967-1987 | 0.240^**^ | |
|  | (0.076) | |
| *=p<.05, **=p<.01, ***=p<.001  Notes: Standard errors in parentheses.  Model includes controls for age, baseline health, race, education, marital status in 1987, children in 1986, an interaction term for time and waves worked, last valid work status, age at first job, and income in 1987. | |  |

| Table A3: Summary of results from regression analyses for functional limitations with “don’t do” responses coded as missing (N=2,763) | |
| --- | --- |
| Proportion waves worked 1967-1987 | -0.573^***^ |
|  | (0.080) |
| Main occupation group 1967-1987 (Comparison: Blue collar) |  |
| White collar | 0.015 |
|  | (0.058) |
| Pink collar | 0.025 |
|  | (0.055) |
| Average hours worked 1967-1987 | 0.002 |
|  | (0.002) |
| Proportion waves reported discrimination 1967-1987 | 0.463^***^ |
|  | (0.094) |
| Average negative job attitude score 1967-1987 | -0.039 |
|  | (0.045) |
| Proportion waves reported lack of job commitment 1967-1987 | 0.258^***^ |
|  | (0.064) |
| *=p<.05, **=p<.01, ***=p<.001  Notes: Standard errors in parentheses.  All models include age, baseline health, race, education, marital status in 1987, children in 1986, interactions for time and waves worked (for depressive symptoms and functional limitations), last valid work status, age at first job, and income in 1987. | |

| Table A4: Summary of results from regression analyses restricting sample to women who were interviewed every wave from 1967-1987 and had non-missing work information | | | |  |
| --- | --- | --- | --- | --- |
|  | Depressive Symptoms  N=2389 | Functional Limitations  N=2389 | Mortality (HR)  N=2680 | |
| Proportion waves worked 1967-1987 | -0.689^**^ | -0.484^***^ | 0.791^*^ | |
|  | (0.257) | (0.088) | (0.093) | |
| Main occupation group 1967-1987 (Comparison: Blue collar) |  |  |  | |
| White collar | -0.157 | -0.028 | .999 | |
|  | (0.178) | (0.064) | (0.093) | |
| Pink collar | 0.187 | 0.013 | 1.074 | |
|  | (0.169) | (0.061) | (0.088) | |
| Average hours worked 1967-1987 | -0.006 | 0.000 | 0.999 | |
|  | (0.005) | (0.002) | (0.003) | |
| Proportion waves reported discrimination 1967-1987 | 1.334^***^ | 0.506^***^ | 1.331 | |
|  | (0.292) | (0.105) | (0.207) | |
| Average negative job attitude score 1967-1987 | 0.434^**^ | -0.038 | 0.921 | |
|  | (0.141) | (0.051) | (0.066) | |
| Proportion waves reported lack of job commitment 1967-1987 | 0.790^***^ | 0.266^***^ | 1.089 | |
|  | (0.198) | (0.071) | (0.096) | |
| *=p<.05, **=p<.01, ***=p<.001  Notes: Standard errors in parentheses.  All models include age, baseline health, race, education, marital status in 1987, children in 1986, interactions for time and waves worked (for depressive symptoms and functional limitations), last valid work status, age at first job, and income in 1987. | | | |  |

| Table A5: Results from regression analyses with time-varying controls for marital status and income | | | |  |
| --- | --- | --- | --- | --- |
|  | Depressive Symptoms  N=2781 | Functional Limitations  N=2781 | Mortality (HR)  N=3207 | |
| Proportion waves worked 1967-1987 | -0.950^***^ | -0.590^***^ | 0.798^*^ | |
|  | (0.245) | (0.083) | (0.086) | |
| Time-varying marital status (Comparison: Never married) |  |  |  | |
| Married | -0.244 | -0.031 | 1.737^***^ | |
|  | (0.309) | (0.109) | (0.250) | |
| Divorced | 0.223 | 0.153 | 2.408^***^ | |
|  | (0.334) | (0.118) | (0.385) | |
| Widowed | 0.420 | 0.054 | 2.181^***^ | |
|  | (0.311) | (0.111) | (0.319) | |
| Time-varying logged family income | -0.558^*^ | -0.199^*^ | 0.986 | |
|  | (0.265) | (0.090) | (0.008) | |
| Main occupation group 1967-1987 (Comparison: Blue collar) |  |  |  | |
| White collar | -0.240 | -0.003 | 0.955 | |
|  | (0.169) | (0.060) | (0.081) | |
| Pink collar | 0.159 | 0.038 | 1.081 | |
|  | (0.160) | (0.057) | (0.081) | |
| Average hours worked 1967-1987 | -0.003 | 0.002 | 0.999 | |
|  | (0.005) | (0.002) | (0.002) | |
| Proportion waves reported discrimination 1967-1987 | 1.296^***^ | 0.476^***^ | 1.285 | |
|  | (0.273) | (0.098) | (0.180) | |
| Average negative job attitude score 1967-1987 | 0.392^**^ | -0.018 | 0.922 | |
|  | (0.131) | (0.047) | (0.060) | |
| Proportion waves reported lack of job commitment 1967-1987 | 0.788^***^ | 0.262^***^ | 1.068 | |
|  | (0.187) | (0.067) | (0.087) | |
| *=p<.05, **=p<.01, ***=p<.001  Notes: Standard errors in parentheses.  All models include age, baseline health, race, education, children in 1986, interactions for time and waves worked (for depressive symptoms and functional limitations), last valid work status, and age at first job. | | | |  |

| Table A6: Summary of results from regression analyses for depressive symptoms and functional limitations with interactions between all work characteristics and time (N=2,781) | | |
| --- | --- | --- |
|  | Depressive Symptoms | Functional Limitations |
| Proportion waves worked 1967-1987 | -0.977^***^ | -0.612^***^ |
|  | (0.263) | (0.086) |
| Main occupation group 1967-1987 (Comparison: Blue collar) |  |  |
| White collar | -0.276 | 0.109 |
|  | (0.199) | (0.066) |
| Pink collar | -0.023 | 0.027 |
|  | (0.191) | (0.063) |
| Average hours worked 1967-1987 | 0.001 | 0.001 |
|  | (0.006) | (0.002) |
| Proportion waves reported discrimination 1967-1987 | 1.350^***^ | 0.485^***^ |
|  | (0.329) | (0.107) |
| Average negative job attitude score 1967-1987 | 0.376^*^ | -0.027 |
|  | (0.158) | (0.052) |
| Proportion waves reported lack of job commitment 1967-1987 | 0.846^***^ | 0.275^***^ |
|  | (0.225) | (0.073) |
| White collar 1967-1987 * Time | 0.027 | -0.067^*^ |
|  | (0.049) | (0.018) |
| Pink collar 1967-1987 * Time | 0.077 | 0.006 |
|  | (0.052) | (0.019) |
| Average hours worked 1967-1987 * Time | -0.002 | 0.000 |
|  | (0.002) | (0.001) |
| Proportion waves reported discrimination 1967-1987 * Time | -0.012 | -0.016 |
|  | (0.086) | (0.032) |
| Average negative job attitude score 1967-1987 * Time | -0.007 | 0.003 |
|  | (0.043) | (0.016) |
| Proportion waves reported lack of job commitment 1967-1987 * Time | -0.035 | -0.014 |
|  | (0.060) | (0.022) |
| *=p<.05, **=p<.01, ***=p<.001  Notes: Standard errors in parentheses.  All models include age, baseline health, race, education, marital status in 1987, children in 1986, interactions for time and waves worked, last valid work status, age at first job, and income im 1987. | | |

| Table A7: Summary of results from regression analyses controlling for the average percent of women in respondents’ field of work in 1966-1967 | | | |  |
| --- | --- | --- | --- | --- |
|  | Depressive Symptoms  N=2781 | Functional Limitations  N=2781 | Mortality (HR)  N=3207 | |
| Proportion waves worked 1967-1987 | -0.881^***^ | -0.590^***^ | 0.803^*^ | |
|  | (0.085) | (0.085) | (0.088) | |
| Main occupation group 1987-1967 (Comparison: Blue collar) |  |  |  | |
| White collar | -0.221 | 0.001 | 0.992 | |
|  | (0.175) | (0.062) | (0.087) | |
| Pink collar | 0.136 | 0.015 | 1.112 | |
|  | (0.173) | (0.062) | (0.091) | |
| Average hours worked 1967-1987 | -0.003 | 0.002 | 1.000 | |
|  | (0.005) | (0.002) | (0.003) | |
| Proportion waves reported discrimination 1967-1987 | 1.325^***^ | .470^***^ | 1.274 | |
|  | (0.276) | (0.098) | (0.179) | |
| Average negative job attitude score 1967-1987 | -0.003 | -0.026 | 0.914 | |
|  | (0.005) | (0.047) | (0.058) | |
| Proportion waves reported lack of job commitment 1967-1987 | 1.325^***^ | 0.253^***^ | 1.074 | |
|  | (0.275) | (0.067) | (0.086) | |
| Average percent of occupation group 1966-1967 female | -0.00 | 0.001 | 0.999 | |
|  | (0.003) | (0.001) | (0.001) | |
| *=p<.05, **=p<.01, ***=p<.001  Notes: Standard errors in parentheses.  All models include age, baseline health, race, education, marital status in 1987, children in 1986, interactions for time and waves worked (for depressive symptoms and functional limitations), last valid work status, age at first job, and income in 1987.. | | | |  |

| Table A8: Summary of results from regression analyses for functional limitations and mortality, controlling for depressive symptoms in 1989 | | |
| --- | --- | --- |
|  | Functional Limitations  N=2781 | Mortality (HR)  N=2894 |
| Proportion waves worked 1967-1987 | -0.558^***^ | 0.810 |
|  | (0.082) | (0.093) |
| Main occupation group 1987-1967 (Comparison: Blue collar) |  |  |
| White collar | 0.042 | 0.972 |
|  | (0.060) | (0.088) |
| Pink collar | 0.040 | 1.111 |
|  | (0.060) | (0.088) |
| Average hours worked 1967-1987 | 0.002^*^ | 0.999 |
|  | (0.002) | (0.003) |
| Proportion waves reported discrimination 1967-1987 | 0.389^***^ | 1.288 |
|  | (0.096) | (0.187) |
| Average negative job attitude score 1967-1987 | -0.054 | 0.897 |
|  | (0.046) | (0.059) |
| Proportion waves reported lack of job commitment 1967-1987 | 0.234^**^ | 1.082 |
|  | (0.066) | (0.090) |
| Depressive symptoms score 1989 | 0.067^***^ | 1.025^***^ |
|  | (0.005) | (0.006) |
| *=p<.05, **=p<.01, ***=p<.001  Notes: Standard errors in parentheses.  All models include age, baseline health, race, education, marital status in 1987, children in 1986, interactions for time and waves worked (for functional limitations), last valid work status, age at first job, and income in 1987. | | |

| Table A9: Summary of results from regression analyses with work measured at age 44-50 | | | |  |
| --- | --- | --- | --- | --- |
|  | Depressive Symptoms  N=2781 | Functional Limitations  N=2781 | Mortality (HR)  N=3207 | |
| Proportion waves worked age 44-50 | -0.534^*^ | -0.253^***^ | 0.965 | |
|  | (0.153) | (0.054) | (0.069) | |
| Main occupation group 1987-1967 (Comparison: Blue collar) |  |  |  | |
| White collar | -0.239 | -0.015 | 0.957 | |
|  | (0.170) | (0.061) | (0.081) | |
| Pink collar | 0.136 | 0.032 | 1.088 | |
|  | (0.161) | (0.061) | (0.081) | |
| Average hours worked 1967-1987 | -0.006 | -0.001 | 1.257 | |
|  | (0.005) | (0.002) | (0.175) | |
| Proportion waves reported discrimination 1967-1987 | 1.263^***^ | 0.422^***^ | 0.998 | |
|  | (0.275) | (0.098) | (0.002) | |
| Average negative job attitude score 1967-1987 | 0.354^**^ | -0.031 | 0.904 | |
|  | (0.132) | (0.047) | (0.058) | |
| Proportion waves reported lack of job commitment 1967-1987 | 0.761^***^ | 0.247^***^ | 1.079 | |
|  | (0.188) | (0.067) | (0.087) | |
| *=p<.05, **=p<.01, ***=p<.001  Note: Standard errors in parentheses.  All models include age, baseline health, race, education, marital status in 1987, children in 1986, interactions for time and waves worked (for depressive symptoms and functional limitations), last valid work status, age at first job, and income in 1987. | | | |  |

| Table A10: Summary of results from regression analyses controlling for involuntary job losses before and after 1967 | | | |  |
| --- | --- | --- | --- | --- |
|  | Depressive Symptoms  N=2781 | Functional Limitations  N=2781 | Mortality (HR)  N=3207 | |
| Proportion waves worked 1967-1987 | -0.870^***^ | -0.573^***^ | 0.780^*^ | |
|  | (0.247) | (0.083) | (0.087) | |
| Main occupation group 1967-1987 (Comparison: Blue collar) |  |  |  | |
| White collar | -0.216 | 0.017 | 0.981 | |
|  | (0.171) | (0.060) | (0.088) | |
| Pink collar | 0.140 | 0.039 | 1.092 | |
|  | (0.161) | (0.057) | (0.086) | |
| Average hours worked 1967-1987 | -0.003 | 0.002 | 0.999 | |
|  | (0.005) | (0.002) | (0.003) | |
| Proportion waves reported discrimination 1967-1987 | 1.319^***^ | 0.469^***^ | 1.262 | |
|  | (0.277) | (0.098) | (0.176) | |
| Average negative job attitude score 1967-1987 | 0.3613^**^ | -0.022 | 1.036 | |
|  | (0.092) | (0.046) | (0.046) | |
| Proportion waves reported lack of job commitment 1967-1987 | 0.768^**^ | -0.022 | 0.896 | |
|  | (0.188) | (0.067) | (0.083) | |
| Involuntary job loss (Comparison: no involuntary job loss) | | | | |
| Before 1967 | 0.015 | 0.018 | 1.028 | |
|  | (0.176) | (0.062) | (0.083) | |
| 1969-1977 | 0.157 | 0.043 | 0.992 | |
|  | (0.141) | (0.050) | (0.069) | |
| 1979-1987 | -0.070 | -0.066 | 0.927 | |
|  | (0.1421 | (0.050) | (0.067) | |
| *=p<.05, **=p<.01, ***=p<.001  Notes: Standard errors in parentheses.  All models include age, baseline health, race, education, marital status in 1987, children in 1986, interactions for time and waves worked (for depressive symptoms and functional limitations), last valid work status, age at first job, and income in 1987. | | | |  |

| Table A11: Regression analyses including controls for the proportion of waves respondents were looking for work or reported an involuntary job loss | | | |
| --- | --- | --- | --- |
|  | Depressive Symptoms  N=2781 | Functional Limitations  N=2781 | Mortality (HR)  N=3207 |
| Proportion waves worked 1967-1987 | -0.873^***^ | -0.575^***^ | 0.794^*^ |
|  | (0.247) | (0.083) | (0.085) |
| Main occupation group 1987-1967 (Comparison: Blue collar) |  |  |  |
| White collar | -0.218 | 0.014 | 0.982 |
|  | (0.171) | (0.061) | (0.084) |
| Pink collar | 0.135 | 0.036 | 1.100 |
|  | (0.161) | (0.057) | (0.082) |
| Average hours worked 1967-1987 | -0.003 | 0.001 | 0.999 |
|  | (0.005) | (0.002) | (0.002) |
| Proportion waves reported discrimination 1967-1987 | 1.303^***^ | 0.462^***^ | 1.278 |
|  | (0.277) | (0.098) | (0.180) |
| Average negative job attitude score 1967-1987 | 0.360^**^ | -0.024 | 0.912 |
|  | (0.132) | (0.047) | (0.058) |
| Proportion waves reported lack of job commitment 1967-1987 | 0.772^**^ | -0.257^***^ | 1.073 |
|  | (0.188) | (0.067) | (0.086) |
| Proportion waves looking for work most of last 2 weeks 1967-1987 | 1.729 | 0.137 | 0.971 |
|  | (2.070) | (0.734) | (1.026) |
| *=p<.05, **=p<.01, ***=p<.001  Notes: Standard errors in parentheses.  All models include age, baseline health, race, education, marital status in 1987, children in 1986, interactions for time and waves worked (for depressive symptoms and functional limitations), last valid work status, age at first job, and income in 1987. | | | |

| Table A12: Summary of results from regression analyses controlling for womens’ transitions from school to work before 1967 | | | |  |
| --- | --- | --- | --- | --- |
|  | Depressive Symptoms  N=2781 | Functional Limitations  N=2781 | Mortality (HR)  N=3207 | |
| Proportion waves worked 1967-1987 | -0.889^***^ | -0.575^***^ | 0.775^*^ | |
|  | (0.246) | (0.083) | (0.087) | |
| Main occupation group 1967-1987 (Comparison: Blue collar) |  |  |  | |
| White collar | -0.235 | 0.020 | 0.982 | |
|  | (0.171) | (0.060) | (0.087) | |
| Pink collar | 0.110 | 0.039 | 1.082 | |
|  | (0.161) | (0.057) | (0.085) | |
| Average hours worked 1967-1987 | -0.003 | 0.002 | 0.999 | |
|  | (0.005) | (0.002) | (0.003) | |
| Proportion waves reported discrimination 1967-1987 | 1.308^***^ | 0.462^***^ | 1.242 | |
|  | (0.275) | (0.098) | (0.171) | |
| Average negative job attitude score 1967-1987 | 0.367^**^ | -0.020 | 1.030 | |
|  | (0.132) | (0.047) | (0.045) | |
| Proportion waves reported lack of job commitment 1967-1987 | 0.751^***^ | 0.250^***^ | 0.890 | |
|  | (0.188) | (0.067) | (0.083) | |
| School-work transition (Comparison: did not work before 1967) |  |  |  | |
| Began full-time work while in school full time | -0.460 | -0.211 | 1.133 | |
|  | (0.307) | (0.109) | (0.178) | |
| Left school and began full-time work in the same year | 0.241 | -0.062 | 1.134 | |
|  | (0.178) | (0.063) | (0.178) | |
| Began work 1 year or more after leaving school full-time | 0.331 | 0.018 | 1.184 | |
|  | (0.191) | (0.068) | (0.107) | |
| *=p<.05, **=p<.01, ***=p<.001  Notes: Standard errors in parentheses.  All models include age, baseline health, race, education, marital status in 1987, children in 1986, interactions for time and waves worked (for depressive symptoms and functional limitations), last valid work status, age at first job, and income in 1987. | | | |  |

| Table A13: Summary of results from regression analyses controlling for the reasons why married women who worked before 1967 left their longest full-time job between school and first marriage | | | |  |
| --- | --- | --- | --- | --- |
|  | Depressive Symptoms  N=1668 | Functional Limitations  N=1668 | Mortality (HR)  N=1918 | |
| Proportion waves worked 1967-1987 | -0.821^*^ | -0.494^***^ | 0.715^*^ | |
|  | (0.318) | (0.105) | (0.108) | |
| Main occupation group 1967-1987 (Comparison: Blue collar) |  |  |  | |
| White collar | -0.205 | 0.011 | 0.923 | |
|  | (0.215) | (0.075) | (0.106) | |
| Pink collar | 0.291 | 0.137 | 1.025 | |
|  | (0.213) | (0.074) | (0.105) | |
| Average hours worked 1967-1987 | -0.002 | 0.002 | 1.000 | |
|  | (0.007) | (0.002) | (0.004) | |
| Proportion waves reported discrimination 1967-1987 | 1.437^***^ | 0.391^**^ | 1.431^*^ | |
|  | (0.346) | (0.120) | (0.238) | |
| Average negative job attitude score 1967-1987 | 0.349^*^ | -0.007 | 1.085 | |
|  | (0.117) | (0.059) | (0.061) | |
| Proportion waves reported lack of job commitment 1967-1987 | 0.811^**^ | 0.164^*^ | 0.847 | |
|  | (0.236) | (0.082) | (0.099) | |
| Reasons left longest job between ending school and first marriage (Comparison: still at this job) | | | | |
| Left job involuntarily | -0.512 | -0.169 | 1.195 | |
|  | (0.466) | (0.162) | (0.267) | |
| Quit job | -0.520 | -0.097 | 0.856 | |
|  | (0.392) | (0.136) | (0.170) | |
| *=p<.05, **=p<.01, ***=p<.001  Notes: Standard errors in parentheses.  All models include age, baseline health, race, education, marital status in 1987, children in 1986, interactions for time and waves worked (for depressive symptoms and functional limitations), last valid work status, age at first job, and income in 1987. | | | |  |
